# Is allergic rhinitis associated with hidden hearing loss in pediatric/adolescent patients? A cross-sectional study

**DOI:** 10.3389/fped.2026.1752238

**Published:** 2026-04-10

**Authors:** Shang Gao, Ping Liu, Huifeng Li

**Affiliations:** 1 Ningde Clinical Medical College of Fujian Medical University, Ningde, China; 2 Department of Otolaryngology, Ningde Hospital Affiliated to Ningde Normal University, Ningde, China; 3 Department of Otolaryngology, The First People’s Hospital Affiliated to Shanghai Jiaotong University School of Medicine, Shanghai, China

**Keywords:** allergic rhinitis, auditory function, cochlear damage, cross-sectional study, hidden hearing loss, pediatric

## Abstract

**Introduction:**

This study aims to investigate the potential impact of allergic rhinitis (AR) on auditory function in children/adolescents and whether AR causes hidden hearing loss.

**Methods:**

This cross-sectional study enrolled children/adolescents (12–18 years) with AR between January 2021 and December 2023. The control group was manually matched. Participants underwent a battery of audiological assessments, including pure-tone audiometry with extended high frequencies, tympanometry, distortion product otoacoustic emissions (DPOAE), electrocochleography, auditory brainstem response, and speech audiometry in noise.

**Results:**

A total of 60 AR patients and 30 healthy controls were included. Patients with AR had significantly worse pure-tone air conduction thresholds in the 9–16 kHz range and lower signal-to-noise ratios (SNR) for DPOAE at frequencies of 6–10 kHz. There were also statistically significant decreases in amplitudes of waves V (0.35 ± 0.06 ms vs. 0.47 ± 0.18 ms, *p* = 0.023) and I (0.25 ± 0.08 ms vs. 0.39 ± 0.07 ms, *p* = 0.009), and increases in amplitude ratios of waves V and I (1.46 ± 0.11 vs. 1.22 ± 0.13, *p* = 0.023) at 80 dB nHL. Furthermore, AR patients had significantly greater summation potential (SP)/action potential (AP) amplitude ratios at 80 dB nHL (0.42 ± 0.12 vs. 0.31 ± 0.08, *p* = 0.000) and worse SNR loss on speech audiometry in noise (5.25 ± 2.99 vs. 2.37 ± 2.04, *p* = 0.001).

**Conclusion:**

AR could lead to hearing impairment in children/adolescents, manifested as hidden hearing loss. These findings underline the importance of thorough auditory evaluations for children/adolescents with AR to detect and treat potential hidden hearing loss.

## Introduction

Allergic rhinitis (AR) is an immune-inflammatory disease of the nasal mucosa characterized by clinical symptoms such as an itchy nose, nasal congestion, sneezing, and watery nasal discharge, mediated by IgE and other related cells following allergen exposure. AR is most commonly observed in children/adolescents, with an incidence ranging between 10% and 40%, which has been increasing in recent years (1).

Although AR does not typically threaten life, it seriously affects health and quality of life. AR is well-known to affect the middle ear due to its nasal involvement. In children/adolescents, the incidence of otitis media associated with AR ranges between 5% and 80% (with an average of 23%) (2). Up to now, it's unclear if AR affects the inner ear in children/adolescents and potentially causes sensorineural hearing loss. It's known that the inner ear generates more immune responses compared to the brain. Immunological activity in the inner ear originates from the endolymphatic sac (ES) (3). Additionally, it has been demonstrated that there is an increase in the number of immune complexes in circulation in Ménière's disease (4). Studies also demonstrated evidence of local antibody production in the perilymphatic zone. When the cochlea encounters an antigen, it creates a locally stimulated response, which can lead to hearing loss (5). However, there was no hearing loss in the hearing study of children/adolescents with AR by M. A. Nursoy et al. Despite this, they still believe that with age, AR may trigger the immune response of the ES and become chronic, which potentially causes hearing loss (6, 7).

It is well known that human outer hair cells (OHCs), inner hair cells (IHCs), afferent nerve (AN) fibers, and the synapses between them work together to maintain normal auditory function (8). An animal study showed that when diffuse cochlear neural degeneration does not change behavioral thresholds, hair cell function must remain normal for this to occur; only once hair cell function is abnormal will the behavioral threshold be elevated (9). Therefore, pure-tone audiometry and otoacoustic emissions are more sensitive to OHC impairment and threshold elevation after OHC dysfunction. However, they are much less sensitive to slightly pathological changes in upper OHCs (i.e., IHCs, auditory nerve fibers, and the synaptic transmission between them) (10). When cochlear synaptic lesions occur, but the outer hair cells' function still remains normal, the hearing loss might easily hide from the normal audiogram, referred to as hidden hearing loss (11).

Previous studies have demonstrated that factors such as noise exposure, aging, and ototoxic drugs can cause cochlear synaptopathy, leading to hidden hearing loss. Hidden hearing loss is a type of hearing deficit where a person has normal results on standard clinical hearing tests (audiograms), yet experiences significant difficulty understanding speech, especially in background noise or complex auditory environments (12). This condition is “hidden” because it cannot be detected by routine pure-tone threshold measurements, which are the standard for diagnosing ordinary hearing loss. Previous animal experiments have shown that cochlear synapses are more fragile than OHCs (13), and cochlear synaptopathy could occur months to years, or even decades, before OHC dysfunction (14). Since AR is a disease with an immunological basis, and chronic ES immune response can affect the auditory function of the inner ear, we initially speculated that a similar situation also exists in AR; that is, children/adolescents with AR may have hidden hearing loss. This study aims to explore whether AR could cause hidden hearing loss in AR children/adolescents.

## Material and methods

### Study design and participants

This cross-sectional study was conducted between January 2021 and December 2023 at the Otorhinolaryngology outpatient department of Ningde Affiliated Hospital of Fujian Medical University. According to the Guidelines for the Diagnosis and Treatment of Allergic Rhinitis, the main clinical manifestations include paroxysmal sneezing, nasal discharge of clear watery fluid, nasal itching, and nasal obstruction (two or more symptoms), lasting for more than 1 h per day (15). Eye symptoms such as itching, tearing, redness, and burning sensations may also be present. Anterior rhinoscopy typically reveals pale nasal mucosa, swollen nasal turbinates, and a clear watery papillary reaction. To confirm the diagnosis of AR, it is necessary to combine clinical manifestations with the results of skin prick tests or serum-specific IgE tests, which provide specific evidence of allergens. AR children/adolescents who met the clinical criteria, had a history of allergies, and positive skin prick test results were recruited as the AR group. AR was the only diagnosed condition in these patients, with other potential comorbidities, such as chronic rhinosinusitis and adenoid hypertrophy (grade III or above), excluded through comprehensive physical and radiological examinations. Healthy children/adolescents without AR and with normal audiometric thresholds (from 0.25 to 8 kHz in both ears) were recruited as the control group. The control group was recruited from friends and companions of the AR group and was manually gender-matched and age-matched for this study.

All AR patients met the following inclusion criteria: (1) normal audiometric thresholds from 0.25 to 8 kHz in both ears, with a tympanogram showing A-type results, (2) native speakers of Mandarin Chinese with unremarkable otoscopic examinations, (3) complete clinical records, (4) sufficient diagnostic data (including nasal coronal CT, middle ear CT, medical examinations, and hearing tests), (5) absence of abnormalities in auricles, dry and unobstructed ear canals, and intact tympanic membranes, and (6) an age range from 12 to 18 years old.

Exclusion criteria for all children/adolescents: (1) history of ear disease (such as tinnitus, dizziness, otitis media, ear tumors, etc.), (2) a clear history of exposure to ototoxic drugs, (3) a history of exposure to prolonged noise, (4) a history of systemic diseases, which could affect hearing (such as hypertension, diabetes, nephropathy, thyroid disease, bronchial asthma, autoimmune diseases, anemia, sleep apnea syndrome, and cervical spondylosis), and (5) a history of infectious diseases that could affect hearing (such as mumps, measles, epidemic cerebrospinal meningitis, scarlet fever, diphtheria, typhoid, rubella, herpes zoster, and syphilis). This study was reviewed and approved by the ethical committee of Ningde Affiliated Hospital of Fujian Medical University (Approval number: #20200105), and written informed consent was obtained from participants' guardians.

### Procedures and data collection

The children/adolescents had not received any AR-related treatments, such as steroids or antihistamines, within 2 weeks prior to their visit. All children/adolescents completed a series of audiological assessments, including pure-tone audiometry with extended high frequencies, tympanometry, distortion product otoacoustic emissions (DPOAE), electrocochleography (ECochG), auditory brainstem response (ABR), and speech audiometry in noise.

### Standard prick test

Sensitization was confirmed by skin prick testing to common aeroallergens (such as house dust mites, pollens, molds, and animal dander), performed on the volar aspect of the forearm using standardized commercial extracts, with a drop of each allergen placed on intact skin and pricked through with a disposable lancet; histamine and saline served as positive and negative controls, respectively, and wheal responses were read after 15–20 min. A positive skin prick test was defined as a wheal diameter of ≥3 mm greater than the negative control, and only patients with at least one positive reaction concordant with their clinical history were included in the AR group.

### Pure tone audiometry

An otorhinolaryngological examination was conducted for each case to exclude potential causes of conductive hearing loss. Pure tone audiometry is a recognized audiological assessment that can be used to assess auditory function, including the equalization, quantification, and localization of hearing loss (16). Audiometric thresholds were obtained using a Madsen Astera audiometer (Gn Otometrics GmbH & Co. Kg, Münster, Germany) (ranging from 250 to 16,000 Hz) in a sound-treated booth with background noise <25 dB (A). Pure-tone air-conduction thresholds were measured with TDH-39 headphones at octave intervals from 0.25 to 8 kHz and with circumaural HDA200 high-frequency headsets at 9, 10, 11.2, 12.5, 14, and 16 kHz. Additionally, pure-tone bone-conduction thresholds were acquired from 0.5 to 4 kHz by placing a Radioear B-71 vibrator over the mastoid process of the temporal bone, according to the standards set by the American National Standards Institute (ANSI) (1977) and the International Organization for Standardization (ISO) standards.

### Tympanometry

The functional condition of the middle ear at 0.226 kHz was assessed using an OTOflex100 middle ear analyzer (Madsen, Denmark). Tympanometry results showed a type A tympanogram with a tympanic pressure of ±100 mmH_2_O and an acoustic amplitude of 0.28–1.67 mL measured in all children/adolescents. Acoustic reflexes were also evaluated.

## DPOAE

DPOAE can reflect OHC function. The pass rate of DPOAE is nearly 100% in healthy individuals with normal OHCs. In the otoacoustic emission test, a DPOAE test is considered passed when the response amplitude at each test frequency exceeds the background noise by at least 6 dB [signal-to-noise ratio (SNR) ≥ 6 dB]. The pass rate is defined as the ratio of the number of ears passing the test to the total number of ears tested. DPOAE testing involves analyzing the signal (DPOAE), noise (NS), and SNR, with SNR commonly used to explain the results. DPOAEs were measured using an otoacoustic emissions instrument (Interacoustics Titan, Denmark) in a sound-treated booth. The frequency separation of the primary tones was f_2_/f_1_ = 1.22, with L_1_ and L_2_ set to 65 and 55 dB SPL, respectively. The f_2_ frequencies were swept from 0.5 to 10 kHz at 0.5, 1, 1.5, 2, 3, 4, 5, 6, 7, 8, 9, and 10 kHz. The acoustic stimulus output of 2f_1_-f_2_ was used to conduct DPOAEs, and the response amplitude, background noise, and SNR were recorded. A response amplitude of at least −10 dB SPL and an SNR of at least 6 dB were considered the threshold for detection at each frequency (17).

## ABR

ABR reflects the integrity of the auditory pathway from the cochlea through the auditory nerve to the brainstem, with its waveform comprising a series of peaks and troughs generated by these sequential neural structures. The most important peaks are waves I, III, and V, with latency and amplitude being the main indicators. Wave I represents the summed activity of the cochlear nerves, including iIHCs, AN fibers, and the synaptic transmission between them. Waves III and V originate from higher centers in the auditory brainstem and midbrain (18, 19). ABR was tested using an evoked potential apparatus (Smart EP, US) in an audiometric test room. All children/adolescents were positioned supine with their eyes closed. The non-inverting electrode, inverting electrode, and ground electrode were placed at the vertex, either the mastoid or the forehead, respectively. The impedance was adjusted to below 5 k*Ω*, with the impedance between pairs of electrodes being <2 k*Ω*. Acoustic stimuli were delivered using ER-3A earphones. The stimuli were 100 μs clicks unilaterally delivered at 80 dB nHL in alternating polarity at a rate of 19.3 Hz. Electrical responses were amplified 100,000X, with the filter bandwidth set between 0.1 and 3 kHz. The window time was 15 ms, and 1,024 sweeps were presented at each recording. The test was repeated twice. The amplitude of waves I and V, the latency of waves I, III, and V, as well as the interval periods of I–III, III–V, and I–V were recorded and analyzed.

## ECochG

ECochG can reflect cochlear neural function and consists of cochlear microphonic (CM), summating potential (SP), and compound action potential (CAP). It is an important method for the supportive diagnosis of Ménière's disease. A recent study shows that the SP/AP ratio in ECochG might help detect hidden hearing loss (20). ECochG was tested using an evoked potential apparatus (Smart EP, US) in an audiometric test room. All children/adolescents lie in a side-lying position with the test ear facing up. The ear canals were prepped by scrubbing with a cotton swab coated in Nuprep. Electrode gel was applied to the cleaned portion of the canal and over the gold foil of each tip of the electrode before insertion. The ground electrode was placed on the forehead, the gold foil (non-inverting electrode) was placed in the test ear canal, and the inverting electrode was placed on the opposite mastoid. The impedance between pairs of electrodes was <2 k*Ω*. Acoustic stimuli were delivered by ER-3A earphones. Stimuli were 100 μs clicks delivered at 80 dB nHL in alternating polarity at a rate of 7.1 Hz. A 55 dB nHL contralateral broadband masker was presented to eliminate contamination of the ABR responses from the contralateral ear. Electrical responses were amplified 100,000× with the filter bandwidth set between 0.1 and 3 kHz. Up to 2,000 sweeps were presented at each recording to enable artifact rejection. The Base, SP, and AP peaks were defined by visual inspection by two observers, one of whom was blinded to the AR groups.

### Speech audiometry

Speech audiometry in noise can assess speech recognition ability and better reflect their communication ability in natural environments. Speech audiometry in noise was assessed using the Bamford-Kowal-Bench Speech in Noise (BKB-SIN) test. The BKB-SIN software system, translated into Chinese by Xi Xin of Beijing 301 Hospital, played a test vocabulary list to both ears simultaneously. The speech stimuli were delivered by TDH-39 headphones to both ears, and the intensity was adaptively changed by 5 dB SNR increments, starting at 70 dB nHL. The software system adaptively adjusted the SNR, and the computer automatically calculated the SNR loss. To prevent distractions during speech testing, a 2-minute break was taken before the presentation of each new list. The SNR loss reflects the subjects' speech discrimination ability in noisy environments. The larger the SNR loss, the worse the speech discrimination ability in noise (21).

### Statistical analysis

SPSS software version 23.0 (SPSS Inc., Chicago, Illinois, USA) was used for all statistical analyses. Continuous variables were tested for normality, and all were found to follow a normal distribution. Therefore, they were expressed as mean ± standard deviation (SD) and compared using a t-test. Categorical variables were expressed as *n* (%) and compared using the chi-square test (*χ*^2^ test). A two-sided *p* < 0.05 was considered statistically significant.

## Results

In the stage of recruiting patients, two patients in the AR group were excluded due to AR combined with sleep apnea syndrome, and one patient was excluded due to AR combined with bronchial asthma. In the control group, one patient was excluded due to acute otitis media. After screening, 60 children/adolescents were recruited, consisting of 33 males and 27 females, with an average age of 15.07 ± 2.3 years (ranging from 12 to 18 years), as the AR group. The control group consisted of 30 children/adolescents, including 17 males and 13 females, with an average age of 14.87 ± 2.2 years (ranging from 12 to 18 years). The demographic information between the AR and control groups showed that the two groups were comparable in sex, age, education level, residential environment, family history of allergies, and pet ownership (all *p* > 0.05) (Table 1).

**Table 1:** Comparison of demographic characteristics between the AR group and the control group.

Parameter	AR group (*n* = 60)	Control group (*n* = 30)	*p*
Sex (boy/girl)	33/27	17/13	0.880
age (years)	15.07 ± 2.3	14.87 ± 2.2	0.730
Education level (high school/below high school)	39/21	14/16	0.090
Residential environment (urban/rural)	41/19	18/12	0.430
Family history of allergies (yes/no)	11/49	2/28	0.140
Pet ownership (yes/no)	23/37	9/21	0.359
Frequency of allergy symptoms (times per week)	3.13 ± 1.35	0	/
Duration of AR (months)	15.58 ± 9.76	0	/
Has undergone desensitization treatment (yes/no)	14/46	0/0	/

AR, allergic rhinitis.

*p* < 0.05 was considered statistically significant.

**Figure 1:**
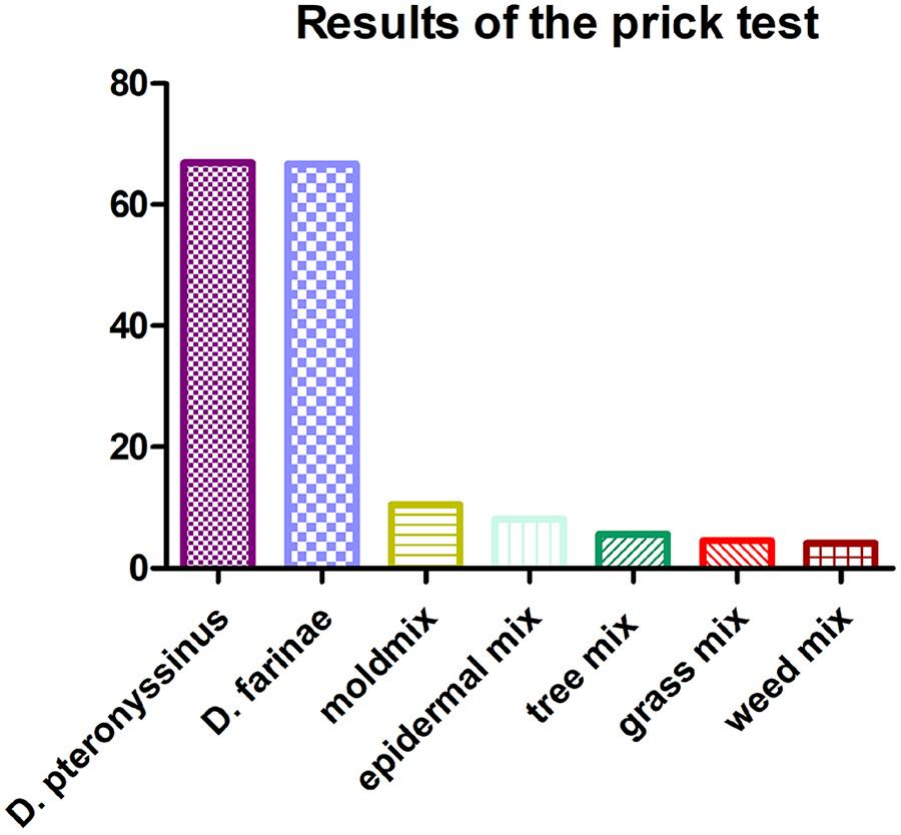
Prick test analysis. The *y*-axis presents the positive prick test results as a percentage of the total numbers.

According to the results of the prick test, the sensitivity of AR children/adolescents to allergens was as follows: *D. pteronyssinus* (66.9%), *D. farinae* (66.7%), mold mix (10.5%), epidermal mix (8.1%), tree mix (5.6%), grass mix (4.6%), and weed mix (4.1%) (Figure 1).

Pure-tone audiometric thresholds of all children/adolescents were assessed. All children/adolescents had normal air conduction thresholds (<20 dB nHL) at the standard audiometric frequencies from 250 to 8,000 Hz, and no statistically significant difference was found between the groups (250 Hz: *p* = 0.810; 500 Hz: *p* = 0.797; 1 kHz: *p* = 0.642; 2 kHz: *p* = 0.563; 4 kHz: *p* = 0.552; 8 kHz: *p* = 0.431). However, above 8 kHz, the AR group may have worse air conduction thresholds at all test frequencies compared with the control group, but some comparisons were borderline and should be interpreted with caution pending confirmation (9 kHz: 11.07 ± 8.36 vs. 9.01 ± 6.64 dB, *p* = 0.048; 10 kHz: 18.62 ± 13.61 vs. 10.97 ± 7.53 dB, *p* = 0.039; 11.2 kHz: 24.14 ± 16.85 vs. 12.58 ± 9.93 dB, *p* = 0.021; 12.5 kHz: 27.22 ± 17.91 vs. 14.93 ± 13.98 dB, *p* = 0.011; 14 kHz: 35.69 ± 17.41 vs. 17.92 ± 15.77 dB, *p* = 0.009; 16 kHz: 36.86 ± 18.50 vs. 19.32 ± 16.73 dB, *p* *<* 0.001) (Table 2).

**Table 2:** Pure tone audiometry at specified frequencies in the AR group and the control group.

Frequency (Hz)	AR group (*n* = 120 ears)	Control group (*n* = 60 ears)	*p*	Mean difference (95% CI)
0.25 kHz	16.53 ± 5.79	15.82 ± 5.31	0.810	0.71 (0.51–0.91)
0.5 kHz	15.33 ± 5.41	15.21 ± 5.34	0.797	0.12 (0.10–0.14)
1 kHz	14.82 ± 4.99	14.75 ± 4.83	0.642	0.07 (0.04–0.10)
2 kHz	13.01 ± 5.24	12.96 ± 5.01	0.563	0.05 (0.03–0.08)
4 kHz	12.97 ± 6.51	11.82 ± 5.89	0.552	1.15 (0.85–1.45)
8 kHz	10.11 ± 2.16	8.80 ± 1.93	0.431	1.31 (0.56–2.06)
9 kHz	11.07 ± 8.36	9.01 ± 6.64	0.048	2.06 (0.61–3.51)
10 kHz	18.62 ± 13.61	10.97 ± 7.53	0.039	7.65 (2.38–12.92)
11.2 kHz	24.14 ± 16.85	12.58 ± 9.93	0.021	11.56 (5.42–17.70)
12.5 kHz	27.22 ± 17.91	14.93 ± 13.98	0.011	12.29 (6.05–18.53)
14 kHz	35.69 ± 17.41	17.92 ± 15.77	0.009	18.77 (7.74–29.80)
16 kHz	36.86 ± 18.50	19.32 ± 16.73	<0.001	27.54 (12.34–42.74)

All are shown as dB nHL. All data are air-conduction thresholds.

AR, allergic rhinitis; CI, confidence interval.

*p* < 0.05 was considered statistically significant. Student's *t*-test.

The SNR of DPOAEs recorded in the AR group was not statistically different between groups across 500 to 5,000 Hz, but the AR group had significantly lower values than the control group across 6,000 to 10,000 Hz (6 kHz: 24.34 ± 6.82 vs. 29.66 ± 4.33 dB, *p* = 0.003; 7 kHz: 24.87 ± 5.55 vs. 29.26 ± 5.10 dB, *p* = 0.004; 8 kHz: 19.63 ± 6.33 vs. 24.43 ± 5.11 dB, *p* = 0.003; 9 kHz: 19.54 ± 8.51 vs. 24.60 ± 5.55 dB, *p* = 0.010; 10 kHz: 16.88 ± 9.73 vs. 22.79 ± 6.73 dB, *p* = 0.011) (Supplementary Figure S1; Table 3). There was no statistically significant difference in the DPOAE pass rate between the AR group and the control group (Table 3).

**Table 3:** The SNR and pass rate of DPOAEs at specified frequencies in the AR group and the control group (dB or pass %).

Frequency (Hz)	AR group (*n* = 120 ears)	Control group (*n* = 60 ears)	*p*	Mean difference (95% CI)
SNR				
0.5 kHz	10.02 ± 4.41	10.20 ± 3.14	0.770	0.18 (0.07–9.29)
1 kHz	17.41 ± 3.37	18.51 ± 5.60	0.411	1.10 (0.47–1.73)
1.5 kHz	21.60 ± 5.69	21.72 ± 5.83	0.743	0.12 (0.06–0.18)
2 kHz	17.35 ± 9.64	21.06 ± 7.61	0.119	3.71 (1.31–6.11)
3 kHz	19.57 ± 4.91	20.02 ± 8.09	0.211	0.45 (0.16–0.74)
4 kHz	21.99 ± 4.15	23.49 ± 7.53	0.316	1.50 (0.97–2.03)
5 kHz	25.56 ± 5.11	27.80 ± 6.29	0.161	2.24 (1.12–3.38)
6 kHz	24.34 ± 6.82	29.66 ± 4.33	0.003	5.32 (2.33–8.31)
7 kHz	24.87 ± 5.55	29.26 ± 5.10	0.004	4.39 (2.16–6.62)
8 kHz	19.63 ± 6.33	24.43 ± 5.11	0.003	4.80 (2.55–7.05)
9 kHz	19.54 ± 8.51	24.60 ± 5.55	0.010	5.06 (2.44–7.68)
10 kHz	16.88 ± 9.73	22.79 ± 6.73	0.011	5.91 (2.88–8.94)
Pass rate				
0.5 kHz	98.33%	98.33%	>0.999	/
1 kHz	100%	100%	0.615	/
1.5 kHz	100%	100%	0.615	/
2 kHz	100%	100%	0.615	/
3 kHz	100%	100%	0.615	/
4 kHz	100%	100%	0.615	/
5 kHz	98.33%	100%	0.315	/
6 kHz	95.00%	98.33%	0.275	/
7 kHz	91.67%	98.33%	0.070	/
8 kHz	90.00%	96.67%	0.115	/
9 kHz	89.17%	93.33%	0.085	/
10 kHz	79.16%	88.33%	0.129	/

SD, standard deviation; AR, allergic rhinitis; CI, confidence interval; SNR, signal-to-noise ratios; DPOAE, distortion product otoacoustic emissions.

*p* < 0.05 was considered statistically significant. The continuous variables are shown as mean ± SD and analysed using Student's *t*-test. The categorical variables are shown as % and were analyzed using the chi-squared test for categorical variables.

There were no statistically significant differences in the latencies of waves I, III, and V between groups at 80 dB nHL, including the inter-peak latencies of waves I–III, III–V, and I–V. However, the amplitudes of waves I and V in the AR group were statistically lower than in the control group (I Wave Amplitude: 0.25 ± 0.08 vs. 0.39 ± 0.07 ms, *p* = 0.009; V Wave Amplitude: 0.35 ± 0.06 vs. 0.47 ± 0.18 ms, *p* = 0.023). The ratio of V/I in the AR group was statistically higher than in the control group (1.46 ± 0.11 vs. 1.22 ± 0.13, *p* = 0.023) (Supplementary Figure S2; Table 4).

**Table 4:** The amplitude (µV, mean ± SD) and latency (ms, mean ± SD) of waves I, III and V at 80 dB nHL in the AR group and the control group.

Parameter	AR group (*n* = 120 ears)	Control group (*n* = 60 ears)	*p*	Mean difference (95% CI)
I-wave latency	1.58 ± 0.08	1.57 ± 0.07	0.801	0.01 (−)
III-wave latency	3.67 ± 0.03	3.63 ± 0.08	0.788	0.04 (0.01–0.07)
V-wave latency	5.63 ± 0.06	5.60 ± 0.05	0.732	0.03 (0.01–0.05)
I–III interwave latency	2.06 ± 0.05	2.05 ± 0.06	0.622	0.01 (−)
III–V interwave latency	1.97 ± 0.07	1.96 ± 0.05	0.513	0.01 (−)
I–V interwave latency	4.05 ± 0.03	4.03 ± 0.06	0.751	0.02 (0.01–0.03)
I-wave amplitude	0.25 ± 0.08	0.39 ± 0.07	0.009	0.14 (0.09–0.19)
V-wave amplitude	0.35 ± 0.06	0.47 ± 0.18	0.023	0.12 (0.07–0.17)
V-to-I amplitude ratio	1.46 ± 0.11	1.22 ± 0.13	0.023	0.24 (0.13–0.35)

SD, standard deviation; AR, allergic rhinitis; CI, confidence interval.

*p* < 0.05 was considered statistically significant. Student's *t*-test for continuous variables.

Further analysis revealed that the amplitude of SP in the AR group was 0.17 ± 0.05 μV, while it was 0.19 ± 0.08 μV in the control group (*p* = 0.325). The AP amplitude was significantly lower in the AR group (0.38 ± 0.09 μV) than in the control group (0.59 ± 0.11 μV) (*p* < 0.001). The SP/AP ratio was statistically greater in the AR group (0.42 ± 0.12 μV) than in the control group (0.31 ± 0.08 μV) (*p* < 0.001) at 80 dB nHL (*p* < 0.05) (Supplementary Figure S3; Table 5).

**Table 5:** SP, AP and the amplitude ratio of SP/AP at 80 dB nHL in the AR group and the control group (μV, mean ± SD).

Parameter	AR group (*n* = 120 ears)	Control group (*n* = 60 ears)	*p*
SP amplitude	0.17 ± 0.05	0.19 ± 0.08	0.325
AP amplitude	0.38 ± 0.09	0.59 ± 0.11	0.000
SP/AP amplitude ratio	0.42 ± 0.12	0.31 ± 0.08	<0.001

SD, standard deviation; AR, allergic rhinitis; SP, summation potential; AP, action potential.

*p* < 0.05 was considered statistically significant. Student's *t*-test for continuous variables.

The SNR loss in speech audiometry was significantly worse in the AR group (5.25 ± 2.99 dB nHL) than in the control group (2.37 ± 2.04 dB nHL), with a mean between-group difference of 2.88 dB (95% CI: 1.41–4.35; *p* < 0.05).

## Discussion

The principal outcome of this study elucidated that AR can result in hidden hearing loss in children/adolescents. Children/adolescents suffering from AR demonstrated poorer auditory performance, specifically worse pure-tone air conduction thresholds in the high-frequency range from 9 to 16 kHz, along with diminished SNRs of DPOAE at frequencies from 6 to 10 kHz. Furthermore, there were notable reductions in wave V and wave I amplitudes and increases in their amplitude ratios in AR children/adolescents when measured at 80 dB nHL. The SP/AP ratio was also found to be higher in AR children/adolescents at 80 dB nHL, and they exhibited greater SNR loss in speech audiometry under noisy conditions.

Children/adolescents in this study exhibited normal audiometric thresholds (from 0.25 to 8 kHz) in both ears, but pure-tone audiometry with extended high frequencies (from 9 to 16 kHz) showed that the threshold of the AR children/adolescents was increased, which was higher than that of the control group, and the difference was statistically significant, similar to the characteristics of hidden hearing loss after noise exposure reported by Liberman et al. (22) This indicates that the inner ear function of AR children/adolescents was indeed compromised, furthermore, it also states that high-frequency audiometry can provide an early warning of hearing impairment, which might inspire the protection and prevention of more serious auditory damage.

A recent study stated that the sensitivity of DPOAE is better than that of standard pure tone audiometry, which could detect potential damage to the cochlea in the early stages and screen patients with early hearing impairment (23). In the present study, the DPOAEs of all children/adolescents were elicited, and there was no statistically significant difference in the SNR of DPOAE between groups across 0.5–5 kHz. However, the SNR of DPOAE in AR children/adolescents was statistically lower than in the control group across 6–10 kHz. This suggests that AR children/adolescents had high-frequency hearing impairment, similar to the characteristics of hidden hearing loss after noise exposure reported in university students by Liberman et al. (22).

In the present cohort, auditory abnormalities were most prominent at extended high frequencies (9–16 kHz on pure-tone audiometry; 6–10 kHz on DPOAE), whereas conventional audiometric frequencies were comparatively spared. This pattern is consistent with experimental and clinical data indicating that the basal, high-frequency region of the cochlea is particularly vulnerable to early damage from metabolic, inflammatory, or noise-related stressors, such that extended high-frequency thresholds often deteriorate before conventional frequencies show measurable change (24, 25). The cochlea is an organ in the inner ear responsible for hearing, with a shape that gradually narrows from base to top. The perception of high-frequency sounds primarily relies on hair cells and nerve fibers at the base of the cochlea, while the perception of low-frequency sounds depends on the top region (26–28). Hence, the vibrations caused by high-frequency sound waves at the base of the cochlea are greater than those at the top, requiring a higher metabolic rate from the hair cells and neurons at the base to process high-frequency signals. Furthermore, the blood supply to the base of the cochlea is relatively poor, making it more susceptible to the effects of chronic diseases, ischemia, oxidative damage, aging, environmental noise, and oxidative stress (29). AR can trigger an immune response in the inner ear, continuously or repeatedly stimulating and interfering with the cochlea, thus causing high-frequency hearing to be affected earlier and more severely in children/adolescents with AR. It aligns with the cumulative damage patterns in biological systems. Extended high-frequency measures and high-frequency DPOAEs are recognized as sensitive indicators of subtle outer hair cell and synaptic dysfunction and have been proposed as early markers of “hidden” cochlear damage. In this context, the preferential involvement of extended high frequencies in children/adolescents with AR suggests that allergen-driven or inflammatory mechanisms may initially compromise the most vulnerable cochlear regions, leading to early high-frequency loss and degraded speech-in-noise performance despite near-normal standard audiometric thresholds (24, 30, 31).

A recent study showed that the SP/AP ratio of ECochG might help to identify hidden hearing loss, which has sparked the interest of many clinicians (20). In addition, with the development of the ear canal gold-foil electrodes in recent years, their applications in clinical work are expected to increase. This present study showed that the SP/AP ratios in AR children/adolescents were significantly greater than in the control group at 80 dB nHL, which further showed that AR children/adolescents had hidden hearing loss.

ABR was used to evaluate the function of the auditory pathways from cranial nerve VIII to the brainstem, which can reflect the auditory function (32). In the present study, the amplitude of wave I in AR children/adolescents was significantly lower than in the control group, which is similar to findings in other studies on ABR with cochlear synaptopathy (33). The amplitude of wave I reflects the summed responses to the primary AN fibers innervating the inner hair cells (IHCs) of the cochlea. Thus, the results of the present study indirectly suggested that the number of responsive AN fibers and the cochlear synchronization in the discharge of AN fibers in AR children/adolescents might be reduced. In addition, the amplitude of wave V in AR was significantly lower than in the control group. Previous studies have shown that waves III and V reflect activity in a pathway originating in the ventral cochlear nucleus (VCN) and especially in spherical bushy cells (SBC) (34, 35). However, there were no statistically significant differences in the wave I-V intervals between groups in the present study. Based on this result, we surmise that the decreases in the amplitude of wave V may be due to a reduction in AN fibers' input originating from the decrease in wave V. Surprisingly, our results showed that the V/I ratios in AR children/adolescents were greater than in the control group. It is well established that waves I, III, and V are generated by a population of consecutively arranged neurons. Beginning with AN fibers, which provide the major source of excitatory input for the SBC, the V/I ratio of the ABR quantifies the extent to which subsequent group activities of the SBC pathway are influenced by decreased AN activity (36). The V/I ratios herein increased in AR children/adolescents, showing that the decreased activity was not only carried forward. Rather, one or more mechanisms transparently work to enhance the population nerve activity of the SBC pathway. Furthermore, animal neurophysiological studies also support the presence of increased SBC excitability following acoustic trauma (36). Therefore, we speculate that AR children/adolescents exhibit different degrees of pathological changes from the cochlea to the inferior colliculus compared with the control group.

The AN fibers of type I innervating the IHCs of the cochlea can be divided into low and high spontaneous discharge rates (low-SR and high-SR) according to the different spontaneous discharge rates (37). Previous studies have shown that low-SR fibers with high thresholds are much more likely to be damaged (38). Damage to low-SR auditory nerve fibers associated with IHCs leads to impaired speech discrimination in noisy environments, while the function of outer hair cells (OHCs) similarly influences speech-in-noise performance and is reflected by reductions in DPOAEs (39–41). When low-SR fibers are damaged, it is often difficult to detect with threshold detection (such as pure tone audiometry), but speech audiometry in noise perhaps could make a contribution; yet such hidden hearing loss is much more common in clinical practice. In this work, the results of speech audiometry in noise showed that the SNR loss of AR children/adolescents was significantly greater than in the control group, which stated that although AR children/adolescents had a normal audiogram, their ability to understand speech in noisy environments was indeed compromised, similar to and correlated with audiometry with extended high frequencies. Those indicated that the low-SR fibers in AR children/adolescents might be damaged.

AR-induced inflammation may affect cochlear neural structures through multiple molecular pathways, including neuroimmune interactions, mediator-induced hair cell damage, and blood-labyrinth barrier disruption. Indeed, AR activates nociceptive neurons via receptors such as TRPV1 and TRPA1, resulting in neuropeptide release (substance P and CGRP). These neuropeptides induce immune cell activation, increased inflammatory mediator secretion, and vasodilation. Histamine and leukotrienes released during AR can interact with C fibers and trigger neurogenic inflammation, altering the sensitivity and function of neurons in both the nasal mucosa and potentially the auditory pathways (42, 43). Experimental data indicate that AR can lead to high-frequency sensorineural hearing loss due to the actions of inflammatory mediators on the inner ear. The endolymphatic sac, which participates in antigen processing and local immune response, may be a target organ, allowing antigens to stimulate mast cell degranulation and accumulation of toxic metabolic products around hair cells. This inflammation is mediated by the deposition of immune complexes in cochlear vasculature, complement activation, and recruitment of inflammatory cells, all of which can disrupt hair cell function and neural signaling (44–46). Allergic reactions may increase vascular permeability by damaging the blood-labyrinth barrier, facilitating the entry of autoantibodies and circulating immune complexes into the inner ear. This results in local inflammation, altered ionic and fluid balance, and direct neural injury, further contributing to cochlear dysfunction and sensorineural hearing loss (44–46). Nasal epithelial cells (NECs) actively communicate with regulatory T cells (Tregs) via chemokines such as CCL1/CCR8. After allergen exposure, cytokines like TSLP, IL-25, and TGF-β are upregulated, facilitating immune cell migration and chronic inflammation. These pathways may contribute to persistent neuroimmune activation and may influence neural cells in adjacent structures such as the cochlea (47).

Maihoub et al. (48) provided a clear example that hydropic inner ear disease can differentially affect cochlear and vestibular function, which conceptually supports the AR/HHL hypothesis. In their comparative study of Ménière's disease, they assessed hearing with pure-tone audiometry and vestibular function with caloric and video head impulse tests and found only a partial relationship between the degree of hearing loss and vestibular impairment, indicating that auditory and vestibular deficits do not necessarily progress in parallel despite a shared hydropic or inflammatory substrate. This aligns with broader evidence in Ménière's and other hydropic ear conditions showing that endolymphatic hydrops may preferentially involve cochlear or vestibular structures in different patients, leading to prominent auditory symptoms with relatively preserved vestibular function in some cases and the opposite pattern in others. By analogy, these observations support the plausibility that allergen-driven inflammatory or fluid-dysregulation processes in children/adolescents with AR could primarily target vulnerable cochlear regions and synapses, manifesting as hidden hearing loss and degraded speech-in-noise performance, while vestibular involvement may be absent, subclinical, or only partially concordant with the auditory findings (49).

The use of a multi-test battery in the present AR/HHL study was an intentional design choice, grounded in contemporary vestibular and audiological research. In disorders such as Ménière's disease and vestibular neuritis, Molnár et al. (50) demonstrated that caloric testing and the video head impulse test exhibit divergent diagnostic profiles, with caloric testing providing higher sensitivity and vHIT higher specificity, and importantly, no meaningful correlation between the two measures. These findings underscore that different tests probe distinct dynamic ranges and anatomical components of inner ear function, so that an isolated test cannot adequately capture the complexity of peripheral auditory and vestibular involvement. By analogy, our AR/HHL protocol was deliberately constructed as a multi-test battery to interrogate complementary facets of inner ear function (e.g., frequency-specific thresholds, supra-threshold processing, and/or vestibular contributions), thereby reducing the risk of underestimating subtle dysfunction. This design is therefore essential and justified, not redundant, and is consistent with current best practice advocating comprehensive test batteries rather than single-test approaches.

In adults, Mahajan et al. (44) and Prabakaran et al. (51) compared hearing outcomes in patients with AR with those in healthy controls, finding that the patients with AR had significantly worse conventional high-frequency hearing than the healthy controls, suggesting that AR can lead to sensorineural hearing loss. However, in the present study, the children/adolescents with AR had normal conventional high-frequency hearing, but their extended high-frequency hearing differed from that of the control group. It may be related to the fact that AR damage to the inner ear may be cumulative over time. Children/adolescents experience the effects of AR for a shorter period than adults, therefore their hearing impairment is milder and more insidious.

This study had several limitations. First, the cross-sectional studies cannot determine causality; additional studies are necessary to determine causality. Second, the moderate sample size and single-center recruitment may limit the external validity and generalizability of our findings. The limited sample size also reduced the statistical power to detect subtle differences in hearing thresholds, particularly in subgroup analyses. Moreover, due to the relatively small sample size, we did not perform multivariable regression analyses to adjust for potential confounders, which may have resulted in residual confounding. A key methodological consideration is the recruitment of the control group from friends and classmates of the AR patients. This strategy was employed to reduce potential confounding by shared living and educational environments (e.g., similar school attendance, daily routines, and local environmental exposures). However, we acknowledge this approach may introduce significant selection bias. For instance, controls might exhibit heightened health awareness or different health-seeking behaviors due to their close relationship with AR patients. They may also share other unmeasured characteristics (e.g., dietary habits, subtle lifestyle factors, or even genetic predispositions) that could systematically differ from the general pediatric population. This potential bias could either inflate or mask the true association between AR and auditory function, and therefore, the results should be interpreted with caution. Additionally, data on intermittent vs. persistent AR were not collected, preventing more in-depth analysis. Although this study conducted several statistical analyses, apart from the comparison of the children's basic information, the audiological test results were in fact all comparisons between two independent groups. In addition, the primary analyses were hypothesis-driven, focusing on predefined exposures/outcomes rather than exploratory screening. Therefore, applying a multiple testing correction to mitigate Type I error (e.g., Bonferroni or FDR) could increase the risk of type II error and obscure potentially meaningful findings. Finally, the significance of the results was carefully interpreted in the context of biological plausibility and consistency with prior evidence, rather than relying solely on nominal *p*-values. As such, we believe that our analytical approach and cautious interpretation adequately address concerns regarding spurious associations. Further research in larger, multi-center, and more diverse cohorts with longitudinal designs is warranted to confirm these findings and to elucidate the underlying mechanisms.

## Conclusion

The present study underscores the importance of recognizing the link between AR and hidden hearing loss in children/adolescents, advocating for a proactive approach to audiological monitoring and care for this group. This could ultimately lead to improved outcomes in terms of hearing health and overall quality of life for affected children/adolescents. Similar analyses should also be performed in adults with AR.

## Data Availability

The original contributions presented in the study are included in the article/Supplementary Material, further inquiries can be directed to the corresponding author.

## Supplementary material

SUPPLEMENTARY FIGURE S1 SNR of DPOAEs at specified frequencies for each group. SNR, signal-to-noise ratios; DPOAE, distortion product otoacoustic emissions.

SUPPLEMENTARY FIGURE S2 The amplitude of waves I, III and V for each group.

SUPPLEMENTARY FIGURE S3 The amplitude SP, AP, and the amplitude ratio of SP/AP at 80 dB nHL. SP, summation potential; AP, action potential.
