# Supplementary figures and images for: Is allergic rhinitis associated with hidden hearing loss in pediatric/adolescent patients? A cross-sectional study
Source: Front Pediatr. 2026 Apr 10;14:1752238. doi: 10.3389/fped.2026.1752238 (PMC13106496; doi:10.3389/fped.2026.1752238)

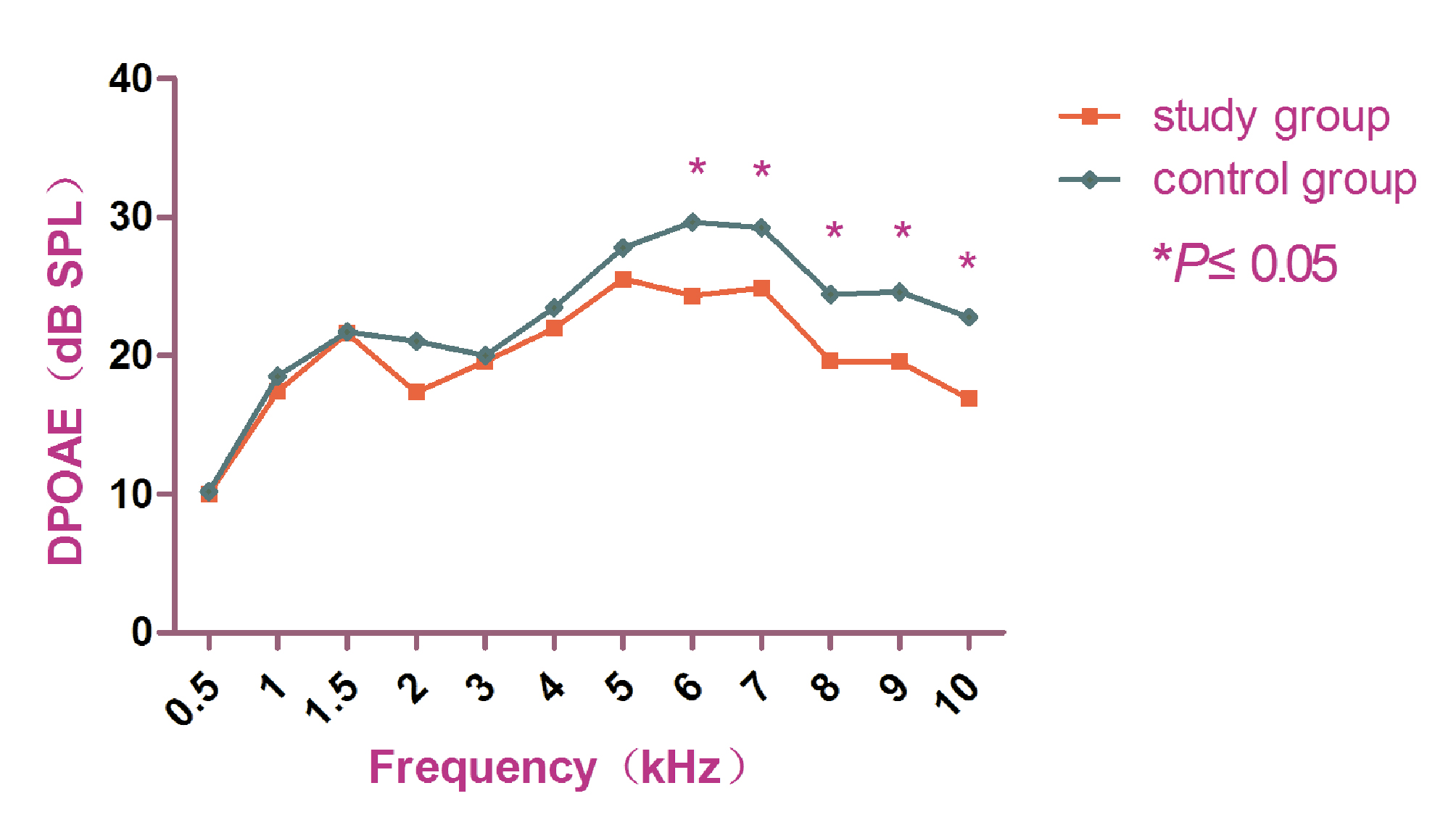

Supplement: SUPPLEMENTARY FIGURE S1 — SNR of DPOAEs at specified frequencies for each group. SNR, signal-to-noise ratios; DPOAE, distortion product otoacoustic emissions. [file Image1.jpeg]

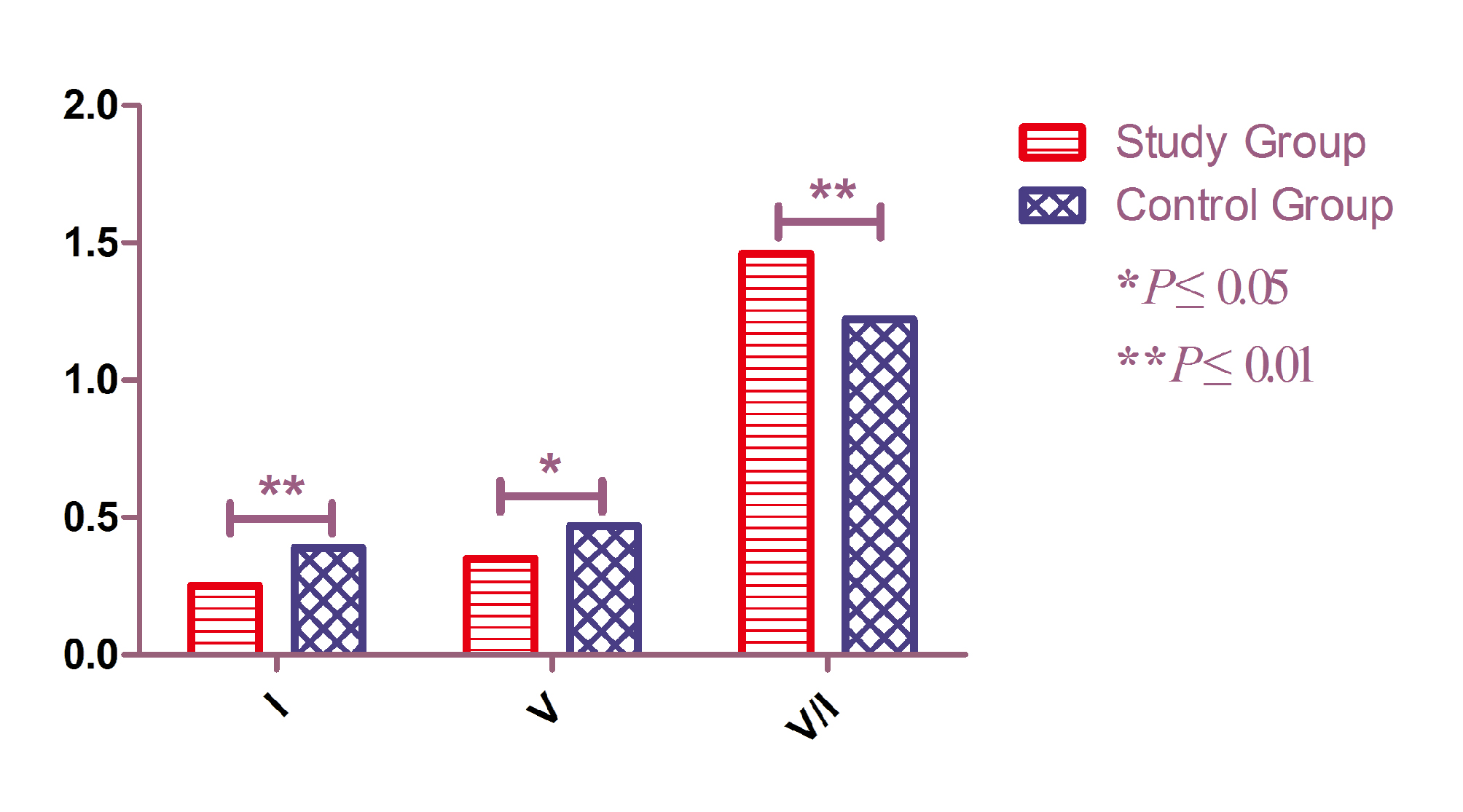

Supplement: SUPPLEMENTARY FIGURE S2 — The amplitude of waves I, III and V for each group. [file Image2.jpeg]

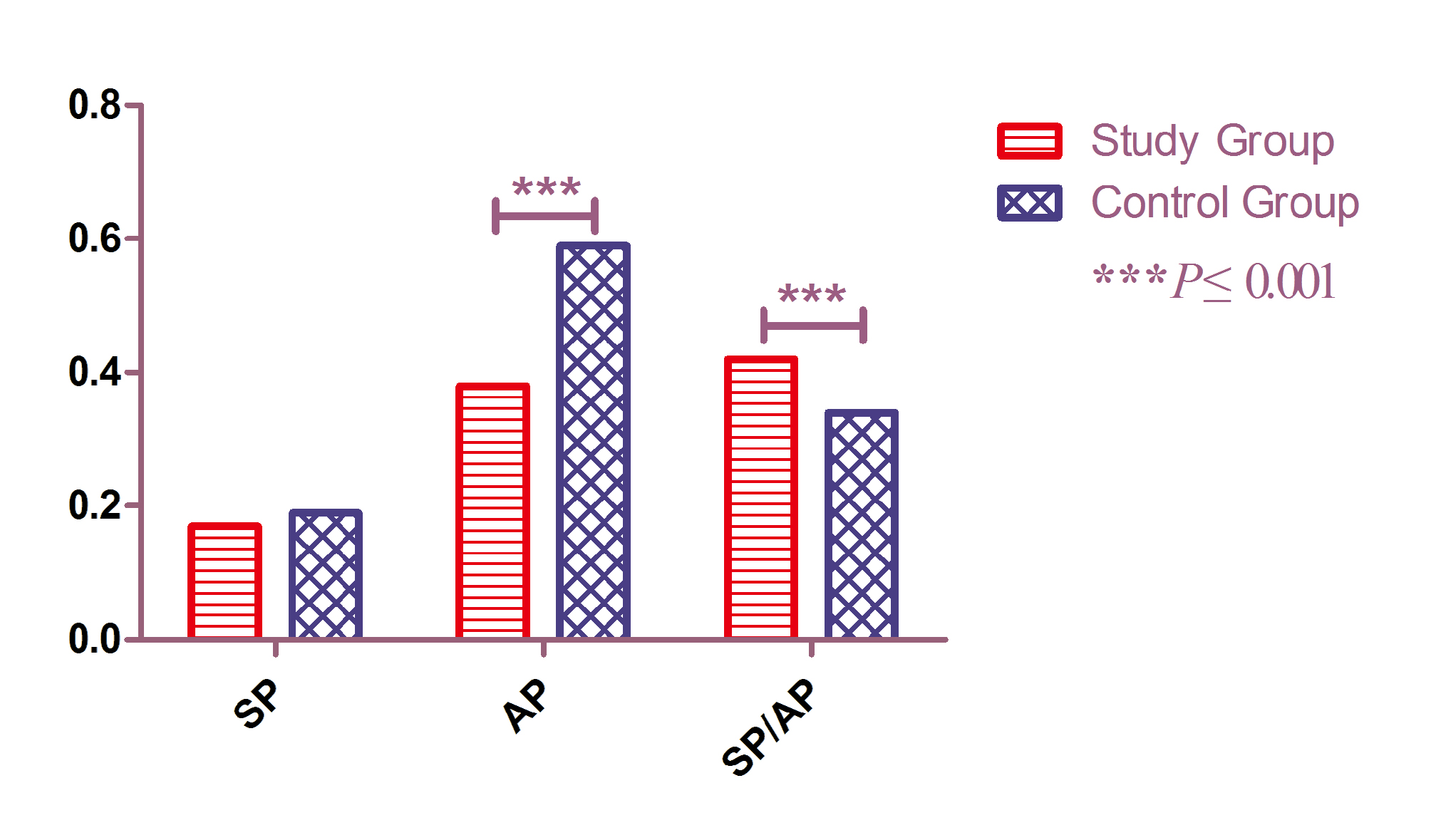

Supplement: SUPPLEMENTARY FIGURE S3 — The amplitude SP, AP, and the amplitude ratio of SP/AP at 80 dB nHL. SP, summation potential; AP, action potential. [file Image3.jpeg]
